# Reporting Digital Health Implementations Based on the iCHECK-DH Guidelines and Checklist: Development of an Interactive Toolkit

**DOI:** 10.2196/74235

**Published:** 2025-08-08

**Authors:** Anuradha Liyanage, Shakira Irfaan, Loshan Moonesinghe, Kaushalya Kasturiaratchi, Caroline Perrin Franck, Rada Hussein

**Affiliations:** 1 Ludwig Boltzmann Institute for Digital Health and Prevention, Lindhofstraße 22, Salzburg, 5020, Austria, 43 05725582701; 2 Ministry of Health, Family Health Bureau, Colombo, Sri Lanka; 3 Faculty of Medicine, Department of Radiology and Medical Informatics, University of Geneva, Geneva, Switzerland; 4 Geneva Digital Health Hub, Geneva, Switzerland

**Keywords:** iCHECK-DH, digital health, toolkit development, guidelines, reporting guideline, implementation science

## Abstract

**Background:**

Despite their potential, many digital health implementations fail to scale beyond pilot stages due to reporting challenges, stakeholder disengagement, and policy barriers. To improve documentation and knowledge sharing, the iCHECK-DH (Guidelines and Checklist for the Reporting on Digital Health Implementations) guidelines have been developed by global experts and implemented by the *Journal of Medical Internet Research* as the required reporting standard for implementation reports.

**Objective:**

This study aims to introduce an interactive iCHECK-DH toolkit designed to streamline reporting, facilitate knowledge sharing, and support scalability, demonstrating its practical application through a use case.

**Methods:**

The iCHECK-DH toolkit was developed through an iterative process informed by best practices for creating user-friendly toolkits. A targeted literature review and analysis of World Health Organization documents were conducted to identify best practices in toolkit design. A multidisciplinary team designed the toolkit using the Fillout platform (Restly, Inc) for its intuitive interface. A total of 9 international experts in digital health and implementation science were recruited through purposive sampling. They provided qualitative feedback through semistructured interviews and open-ended comments on the draft toolkit. Discussions focused on content relevance, usability, and alignment with the iCHECK-DH framework. Feedback was thematically analyzed and key suggestions were incorporated iteratively into subsequent versions of the toolkit. It underwent pilot testing, with a real-world Family Planning Stock Management System in Sri Lanka serving as the use case. The findings further refined the toolkit’s practicality and informed final improvements.

**Results:**

The interactive toolkit successfully translated the iCHECK-DH guidelines into a structured digital health implementations reporting tool, refining navigation, terminology, and usability based on qualitative feedback from the expert panel. It features 4 main sections, including a step-by-step checklist aligned with the 20 items of the iCHECK-DH framework, contextual explanations and examples for each item, a self-assessment function to track progress, and automatic compilation of a downloadable PDF report. Pilot testing showed that the toolkit enabled comprehensive documentation of all 9 domains and 20 checklist items from the iCHECK-DH framework. It confirmed its practicality and effectiveness, leading to targeted improvements. Time to complete the toolkit was 40 minutes (final version) after usability refinements.

**Conclusions:**

The iCHECK-DH toolkit complements the iCHECK-DH guidelines, enhances their functionality, and demonstrates their usability through a practical use case. It provides a structured approach to digital health reporting, supporting scalability and knowledge sharing in real-world implementations.

## Introduction

Digital health technologies have the potential to enhance health care access, efficiency, and patient outcomes [1-3]. In the past few years, the urgency to scale digital health implementations has increased, especially following the COVID-19 pandemic, which underscored the need for resilient and scalable digital infrastructures [4-6]. However, many implementations fail to scale beyond pilot stages rather than expanding into broader, scalable implementations.

This limitation often occurs because pilots do not provide sufficient evidence of effectiveness or value to justify wider adoption [4]. In addition, several implementation challenges, such as provider resistance, insufficient stakeholder engagement, and policy-related barriers, often remain unaddressed during the pilot phase [7-9]. In addition to these challenges, the ability to scale digital health technologies is often hindered by the lack of readiness within health systems and organizations. Successful scaling requires more than just a proven technology; it demands strong leadership, political will, and an infrastructure that can support widespread adoption [7,9]. In many cases, limited workforce capacity, technological fragmentation, and inadequate funding models contribute to the failure of scale-up efforts [4,8].

A key challenge is the lack of standardized documentation, making it difficult to learn from previous implementations, assess impact, and replicate successful interventions [10]. Studies have shown that structured documentation not only improves transparency but also supports strategic decision-making, fosters stakeholder engagement, and increases the chances of sustainability [7,8,11]. As digital health continues to expand, embedding rigorous reporting and evaluation practices into the early stages of implementation will be crucial for translating small pilots into sustainable, large-scale programs. Recent evaluations of digital health programs highlight that inadequate reporting continues to hinder learning, replication, and adaptation across settings [12,13]. Several frameworks have emerged to improve reporting and evaluation, such as the Consolidated Standards of Reporting Trials of Electronic and Mobile Health Applications and Online Telehealth (CONSORT-EHEALTH) [5], the mHealth Evidence Reporting and Assessment (mERA) checklist [14], yet no guidelines supporting the reporting of digital health implementations and processes [11,15].

To address this, a global expert panel developed the iCHECK-DH, a standardized framework ensuring comprehensive reporting of digital health implementations [16]. These guidelines help to ensure that digital health implementations are comprehensively documented by identifying the minimum set of information required. By promoting thorough documentation and dissemination of findings, these guidelines aim to bridge the gap between implementation research and real-world practice, ultimately facilitating the successful deployment of digital health interventions [16].

The iCHECK-DH guidelines were developed using the methodology outlined by Moher et al [17], resulting in a final list of 20 items, which were then tested in workshops and applied to real-world implementations to ensure their effectiveness.

**Figure 1.**
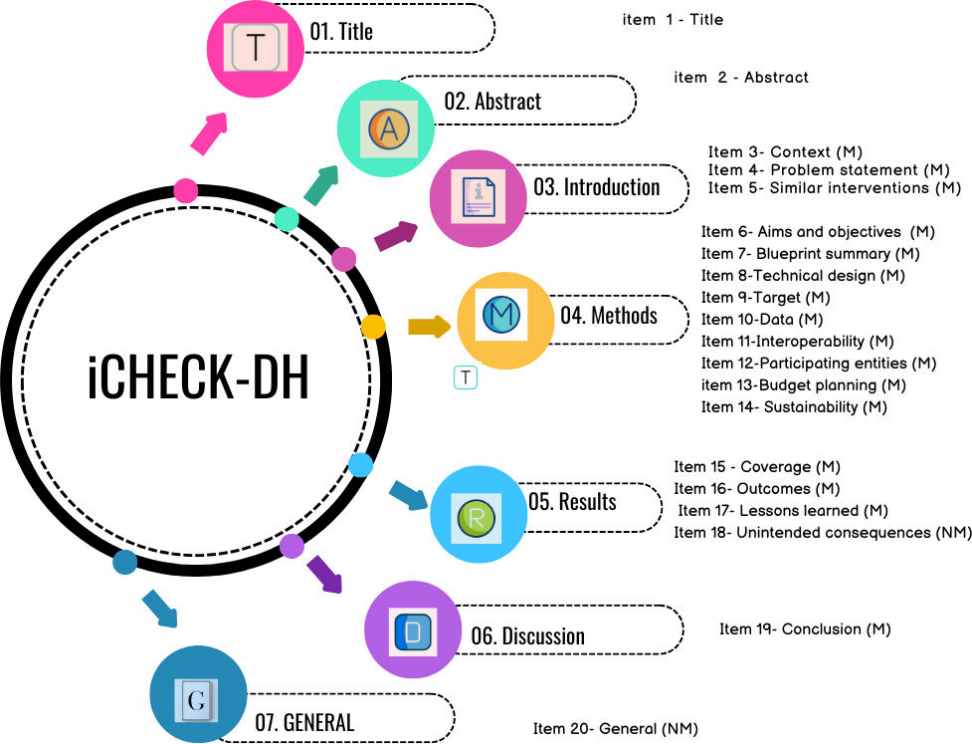
Sections and corresponding items of the iCHECK-DH (Guidelines and Checklist for the Reporting on Digital Health Implementations).

These 20 items are organized into 7 sections: Title (item 1), Abstract (item 2), Introduction (items 3‐5), Methods (items 6‐14), Implementation Results (items 15‐18), Discussion (item 19), and General (item 20). Each item is classified as either mandatory or nonmandatory, with mandatory items representing essential information that authors must provide (Figure 1). Detailed explanations and examples accompany each item to guide users in effectively reporting digital health implementations [16]. Using iCHECK-DH guidelines to report digital health implementations is now required for implementation reports at the *Journal of Medical Internet Research* publication [9,18].

While these guidelines provide structured documentation, practical application remains challenging. The aim of this study was to develop an interactive toolkit that simplifies the process of reporting digital health implementations, making it more interactive, easier, and more efficient for users to apply the guidelines.

## Methods

### Overview

First, a literature review was conducted to explore how a guideline can be effectively translated into a user-friendly toolkit [11,19]. This review included an analysis of best practices from successful toolkits across various domains [11,19,20].

We searched 3 electronic databases PubMed (MEDLINE), EMBASE, and Google Scholar for literature published between 2000 and 2024. Search terms included combinations of “digital health,” “guideline implementation,” “interactive toolkit,” “checklist design,” and “usability.” We also reviewed references of key articles to identify additional relevant sources.

Articles were included if they discussed the development or implementation of digital toolkits, checklists, or interactive frameworks, particularly those related to health or clinical guidelines. Exclusion criteria included papers that focused solely on clinical outcomes, lacked any digital or interactive component, or were editorial or opinion pieces. A total of 9 relevant articles were included, and key findings were synthesized to guide the structure and functionality of the toolkit we developed.

**Figure 2.**
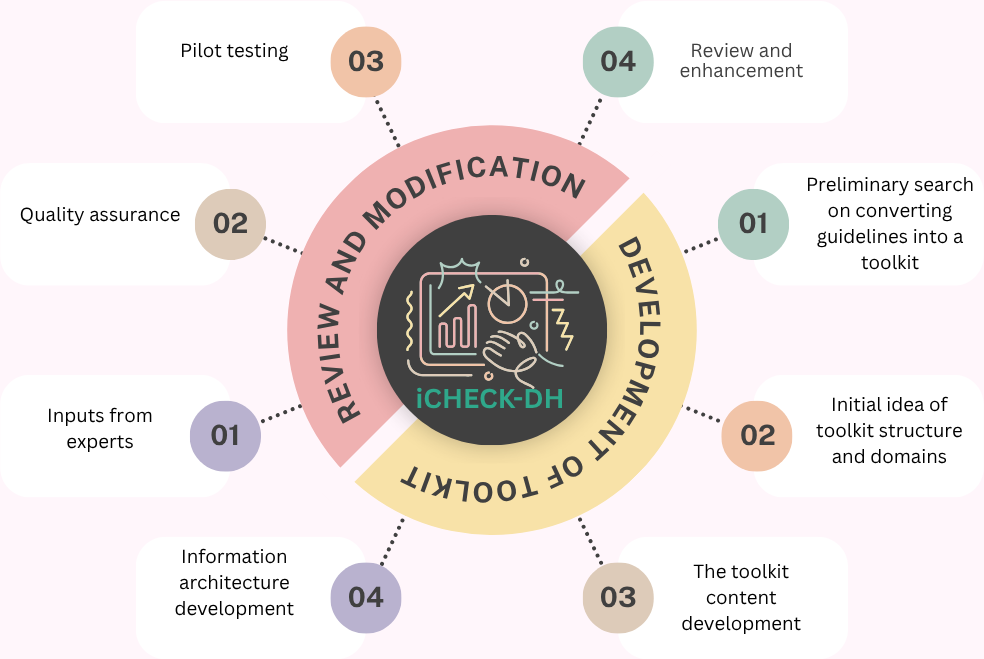
Steps of the iCHECK-DH (Guidelines and Checklist for the Reporting on Digital Health Implementations) toolkit development.

Based on these insights, key steps for developing a high-quality toolkit were identified, using a thematic synthesis of the literature review findings. Concepts extracted from the included studies were categorized by identifying recurring themes such as modular design, user-centered navigation, alignment with reporting standards, and iterative quality and usability testing. These themes were then mapped to specific actions required for toolkit development, including defining content domains, enabling user interaction, conducting expert reviews, performing quality checks, and carrying out pilot testing. An iterative process was agreed upon within the research team consisting of physicians, researchers, public health experts, and health informatics experts to guide the development. The synthesized actions formed the foundation of the iterative development process outlined in Figure 2.

The iCHECK-DH guideline [16] served as the primary reference for developing the content and structure of the toolkit. In addition, other relevant World Health Organization (WHO) documents [21-23] were reviewed to extract standardized definitions, terminologies, and complementary concepts to ensure consistency and alignment with global digital health frameworks. This supplementary review supported the accurate contextualization of key terms and usability aspects within the toolkit. In addition, different platforms suitable for the development of the toolkit were explored and their features and suitability were evaluated.

Fillout was selected as the platform for developing the toolkit due to its user-friendly interface and robust capabilities. The main domains of the toolkit were identified, mapped, and structured to ensure a logical content organization and efficient navigation. Subtopics were determined, and the architecture was drafted to enable seamless navigation and easy cross-referencing. The content was aligned with the iCHECK-DH (Guidelines and Checklist for the Reporting on Digital Health Implementations) [9], which served as the primary reference.

A panel of experts reviewed the draft toolkit and discussions were conducted to refine the approach. The experts included members of the institutional project team and the Scientific Advisory Board of the Ludwig Boltzmann Institute, all with a minimum of 5 years of professional experience in relevant fields. The group comprised 9 specialists in health informatics, human-computer interaction, implementation science, research, public health, and clinical medicine. To minimize potential bias, none of the reviewers was directly involved in the technical development of the toolkit.

Feedback was collected during institutional meetings and dedicated review sessions. Experts were guided through the toolkit prototype and engaged using a semistructured interview guide focused on 4 domains: clarity and appropriateness of content, usability and navigation, alignment with iCHECK-DH principles, and practical recommendations for improvement. Discussions were facilitated by 2 independent researchers and documented through detailed notes and recordings (with consent). Thematic content analysis was used to synthesize feedback and iterative updates to the toolkit were made based on group consensus. This structured and transparent approach supports the reproducibility of the procedure and increases the credibility of our results.

A quality assurance process was conducted to ensure accuracy and usability. The content was validated against the iCHECK-DH guidelines and navigation features were tested for a smooth user experience. Usability testing with 2 independent health informatics experts outside the research team identified challenges, which were documented and addressed to further refine the toolkit.

### Pilot Testing

Pilot testing was conducted by applying the toolkit to an actual digital health implementation, named “Web-based Family Planning Stock Management System” (WFPSMS), Sri Lanka. This testing assessed the effectiveness and practicality of the toolkit in a real-world scenario. Feedback from this pilot application identified remaining gaps and areas for improvement, which were addressed to refine the toolkit (Multimedia Appendix 1).

WFPSMS serves as a centralized, real-time digital platform for managing family planning commodity stocks, ensuring equitable distribution, and enhancing decision-making, replacing the previous paper-based system. It features web-based stock returns and Geographic Information System mapping enabling real-time monitoring of contraceptive availability at all levels (Multimedia Appendix 1).

### Ethical Considerations

As of December 2024, we conducted the pilot test. This study did not involve human participant research as it is based on secondary data analysis. An expert review was conducted, and consent was obtained from the experts involved in the review process. Since the study used secondary data, the original data collection was conducted with informed consent, and the primary research, from which the data were derived, included appropriate consent for the use of data for secondary analyses, in line with institutional review board approval. No personal data were used in this study. As such, there are no concerns regarding confidentiality or privacy for the individuals involved in the original data collection. The study did not include any images of individual participants, and no issues regarding participant identification arose.

## Results

### iCHECK-DH toolkit

**Figure 3.**
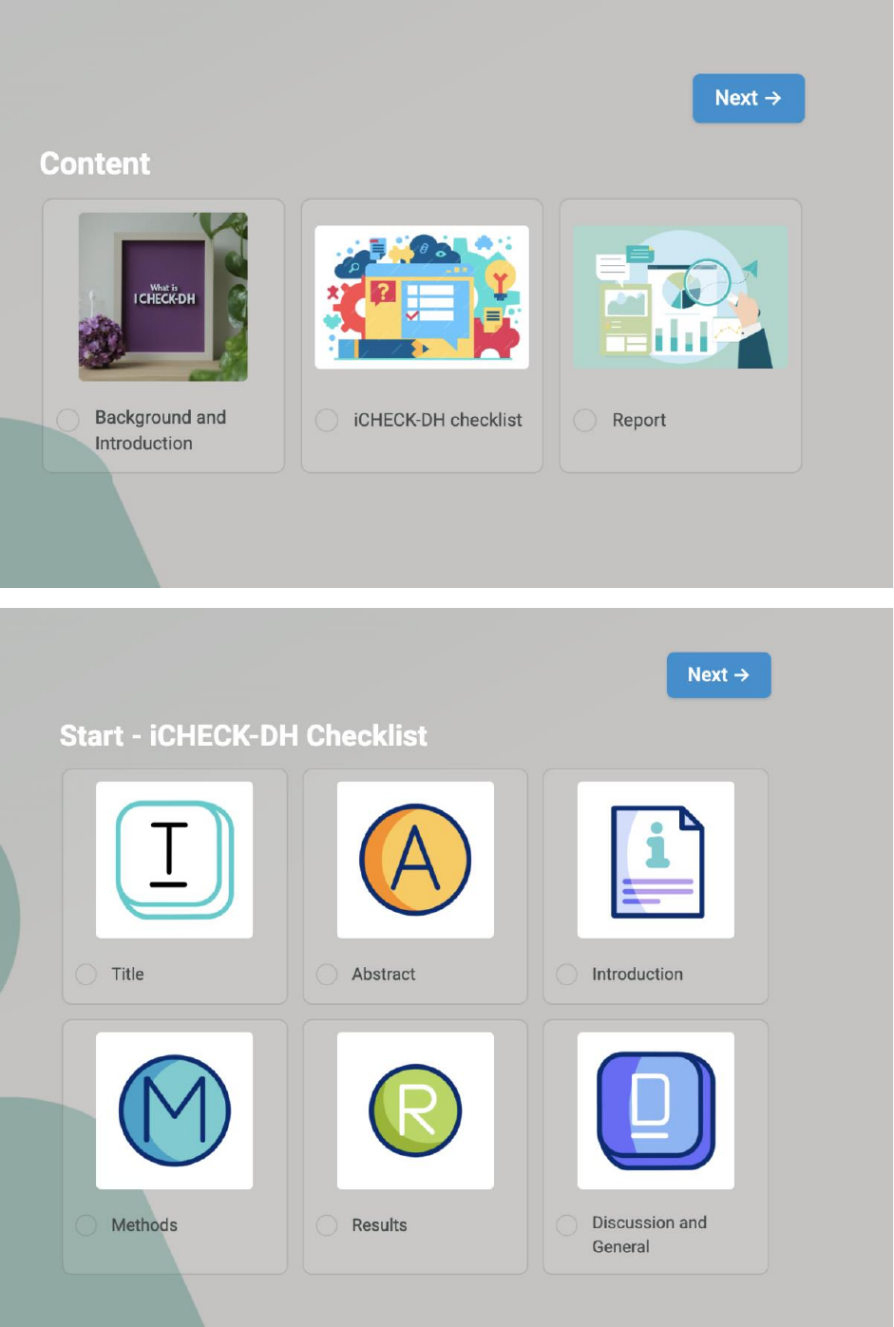
Domains of the iCHECK-DH (Guidelines and Checklist for the Reporting on Digital Health Implementations) toolkit.

The iCHECK-DH toolkit has been carefully designed to ensure clarity and accuracy (Figure 3). It is divided into 3 main sections: Background and Introduction, iCHECK-DH Toolkit, and Report. The toolkit is accessible online [24].

The Background and Introduction section defines key digital health implementation concepts, introduces the toolkit, and provides usage guidance. The iCHECK-DH checklist is structured into 7 sections: Title (Item 1), Abstract (Item 2), Introduction (Items 3‐5), Methods (Items 6‐14), Implementation/Results (Items 15‐18), Discussion (Item 19), and General (Item 20). These 20 items are displayed across 6 sequential pages within the toolkit (Figure 3).

Following the iCHECK-DH guidelines, checklist items are categorized as mandatory or nonmandatory, with clear indications in the toolkit [9]. Mandatory items contain essential information that must be provided by the authors of the intervention. Each item includes contextual explanations and examples to enhance usability.

The toolkit also features a self-assessment function, allowing users to track progress, flag incomplete sections, and revisit them later. All user inputs across sections are automatically compiled into a report. Items flagged as incomplete by the user during self-evaluation are also highlighted in the report. The final report can be downloaded as a PDF or emailed to a specified address, ensuring seamless documentation and information sharing.

**Figure 4.**
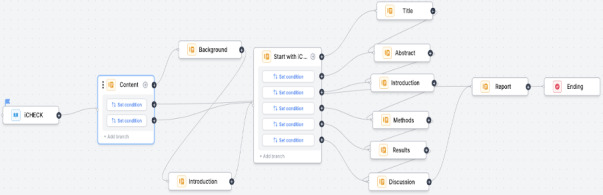
Navigation architecture among interfaces of the toolkit.

The toolkit’s logical and intuitive framework organizes content hierarchically, ensuring ease of navigation and accessibility for users (Figure 4).

A panel of experts reviewed the draft toolkit, providing feedback on content usability and structure. A key recommendation was to allow users to enter responses directly rather than using only a checklist. An expert explained:

*The current structure is clear, but it would benefit from allowing users to input answers directly into the toolkit instead of working separately on a checklist.*

In response, a designated input space was added to enhance usability.

As one expert stated,

*More explanatory text and examples would be helpful, especially for users who are unfamiliar with reporting digital health projects.*

Another expert pointed out,

*The interface is intuitive overall, but the terminology in some sections could be simplified to be more accessible for non-technical users*.

while another added,

*Including a user guide or onboarding tutorial would make the toolkit easier to adopt, particularly for first-time users*.

Accordingly, improvements, including navigation clarity and terminology alignment, were implemented by reorganizing the interface. A user guide, including step-by-step instructions, was also added to the toolkit.

Experts found the toolkit to be highly effective for reporting on digital health implementations, particularly valuing its summary report generation for researchers. Additional improvements included optimized navigation, expanded explanatory content, and a more comprehensive user guide, making the toolkit more intuitive and adaptable for both novice and experienced users.

### Pilot Study

**Figure 5.**
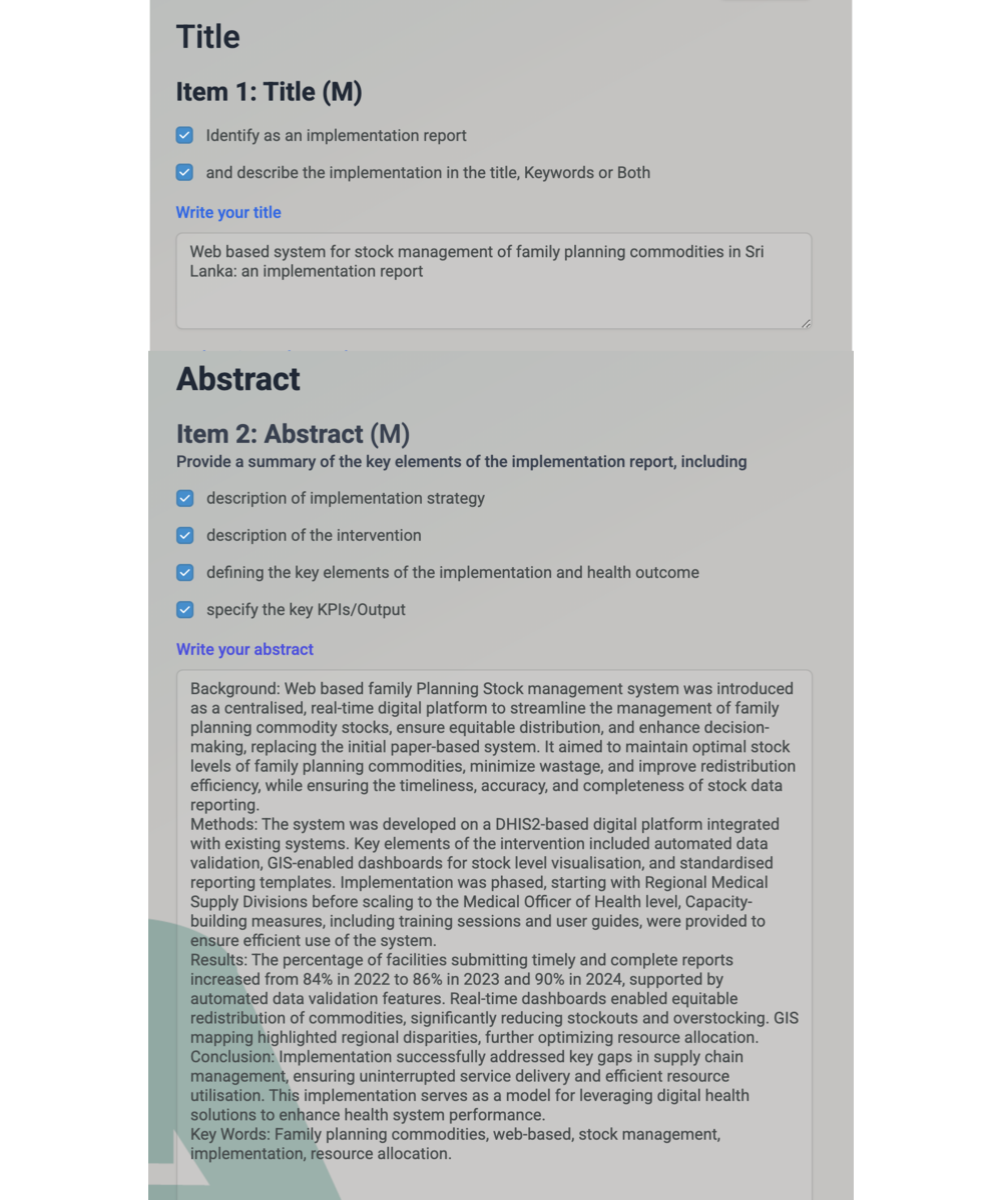
Section 1 and 2 of the implementation report of the Web-based Family Planning Stock Management System were written using the iCHECK-DH toolkit.

The pilot study involved 3 participants who used the iCHECK-DH toolkit to document the WFPSMS in Sri Lanka. The generated implementation report summary included all required sections: Title, Abstract (Figure 5), Introduction, Methods, Results, Discussion, and General sections.

Implemented nationwide, WFPSMS targets health care professionals managing family planning stocks, including Regional Medical Supply Division officers and Medical Officer of Health staff. Developed in-house by information technology specialists and community physicians from the Family Health Bureau, it operates within the electronic reproductive health management information system (eRHMIS), Sri Lanka’s maternal and child health data system. The DHIS2 platform (University of Oslo), a free and open-source solution, was chosen to avoid additional costs.

The phased implementation began at the Regional Medical Supply Division level before expanding to Medical Officer of Health facilities. Comprehensive training sessions and periodic refresher courses ensured continued user engagement. Ongoing system monitoring promotes data use at all levels, fostering evidence-based decision-making and strengthening Family Planning Stock Management in Sri Lanka (Multimedia Appendix 1).

Participants found the toolkit user-friendly and effective for its intended purpose. One noted,

*The toolkit was very easy to complete because it was well-structured and intuitive, ensuring we didn’t miss any critical information during reporting.*

Another highlighted its clarity, saying

*We didn’t initially have a clear understanding of how an implementation should be reported, but the iCHECK-DH toolkit provided exactly that guidance*.

The step-by-step structure simplified adherence to the guidelines. As one participant stated,

*Since the toolkit presents each criterion separately, it didn’t take much effort to follow the guideline.*

The integrated examples and explanations were particularly appreciated:

*It’s great that examples and explanations appear right when needed—it allows us to refer to them easily whenever we require assistance.,*

while another added,

*Reading the examples gave us a clear idea of how to proceed*.

Participants valued the summary report feature, commenting,

*The ability to generate a summary at the end is incredibly useful. The fact that it can be emailed or downloaded as a PDF is an excellent feature. It would be even better if the summary could also be downloaded as a Word document as we can edit later*.

The progress-tracking Likert scale from 0 to 100 was also praised.

*This feature is really useful—it reminded us of which sections we needed to improve. It also allowed us to move ahead with gaps and return later to fill them*.”

Despite the positive feedback, areas for improvement were noted. One participant suggested,

*The colour contrast could be better to enhance visibility.*

Another noted initial confusion about the toolkit’s purpose, suggesting,

*It was a bit unclear at the beginning what we were supposed to do with this toolkit. It would be helpful to have a clear purpose outlined on the first page for someone seeing it for the first time.*

Navigation enhancements were also proposed:

*If there were a top menu to show where we are at any given point, it would make navigation much easier.*

To address the feedback, refinements were made. Color contrast was improved, an introductory page was added, and a top menu was integrated to enhance usability. Furthermore, iterations will explore generating reports in Word format alongside PDFs to better meet user needs. These updates aim to make the toolkit even more accessible for diverse stakeholders. Time to complete the toolkit was 40 minutes (final version) after usability refinements.

## Discussion

### Principal Findings

The development of the interactive iCHECK-DH toolkit represents a major step in improving documentation to facilitate the scaling of digital health implementations. By transforming the iCHECK-DH framework into an accessible, interactive format, the toolkit bridges the gap between theoretical guidelines and practical application. It enhances digital health reporting by supporting comprehensive documentation of context, interventions, and implementation processes, facilitating knowledge sharing, identifying success and failure factors, and connecting research with practice. Standardized reporting supports better practices and scalable digital health solutions.

Previous studies have highlighted that inconsistent reporting impedes replication and cross-country learning in digital health [25]. Our findings support these concerns: participants emphasized that the toolkit’s structured approach made it easier to capture key information—especially context and implementation barriers—that are often omitted in project documentation.

Expert and user feedback played a key role in shaping the toolkit’s functionality. Notable additions included direct response input, onboarding materials, and expanded exploratory content, ensuring usability even for those with limited digital experience.

Pilot testing revealed both strengths and usability gaps. Navigation challenges led to interface improvements, while terminology standardization and accessibility enhancements—such as improved color contrast—increased ease of use. These align with usability standards advocated by the WHO Digital Implementation Investment Guide [23]. Participants praised the summary feature, highlighting its clear, downloadable report format, which improved engagement. This aligns with prior evaluations of similar digital reporting tools that found autogenerated summaries improved both data reuse and stakeholder communication [26].

Future developments could incorporate interactive elements and ongoing user testing to drive continuous improvements. The pilot underscored the importance of user feedback in refining the toolkit to support effective and inclusive digital health solutions. Continuous user testing and iterative design cycles should remain central to future development, consistent with user-centered design principles shown to be effective in digital health tool development [13,27].

The toolkit is and will remain freely accessible through a dedicated URL. Fillout allows for indefinite public publishing under their current service model. We have secured a long-term institutional account to ensure uninterrupted availability. Ongoing management and maintenance of the toolkit will be handled by the digital health and research group at Ludwig Boltzmann Institution. We have embedded a feedback form directly within the toolkit to allow users to report usability issues, content gaps, or suggest improvements. Feedback is reviewed quarterly, and the toolkit will be updated accordingly in line with new developments in the iCHECK-DH framework.

### Limitations

While the pilot testing with the Family Planning Stock Management System in Sri Lanka offered valuable, real-world feedback, we acknowledge that findings from a single vertical health program in a specific country context may not fully capture the needs of other digital health implementations—especially those operating in different health care domains (eg, mental health, chronic disease management, and maternal and child health), different scales (eg, national-scale systems and small pilot apps), and varied resource settings (eg, low-bandwidth and low-literacy environments). We plan to apply it to AktivPlan (Ludwig Boltzmann Institute for Digital Health and Prevention [LBI-DHP]), a digital intervention aimed at supporting health care professionals and patients in collaboratively managing heart-healthy physical activity plans. AktivPlan functions as both a web and mobile app and is used by clinicians to help patients set, monitor, and adjust personalized goals, with ongoing review at follow-up appointments. This second use case focused on cardiovascular disease prevention and self-management will allow us to assess the toolkit’s usability and reporting support in a different health care setting, targeting preventive care in high-income contexts.

Looking ahead, there are several opportunities for enhancing the toolkit further. Expanding multilingual support and collaborations with governments, academic institutions, and digital health organizations could also support the continued refinement and scaling of the toolkit. These partnerships could help integrate the toolkit into broader health systems and policies, increasing its adoption and impact.

### Conclusions

The iCHECK-DH toolkit simplifies digital health reporting by operationalizing a complex framework into a practical and accessible tool. Improving documentation enhances reproducibility and facilitates knowledge sharing in global digital health implementations. While there are opportunities for further enhancement, the toolkit lays a strong foundation for streamlining digital health workflows and ensuring consistent, high-quality reporting across diverse settings.

## Supplementary material

10.2196/74235 Multimedia Appendix 1 Use case report generated by iCHECK-DH (Guidelines and Checklist for the Reporting on Digital Health Implementations) toolkit.

## Abbreviations

CONSORT-EHEALTH: Consolidated Standards of Reporting Trials of Electronic and Mobile Health Applications and Online Telehealth
eRHMIS: electronic reproductive health management information system
iCHECK-DH: Guidelines and Checklist for the Reporting on Digital Health Implementations
WFPSMS: Web-based Family Planning Stock Management System
WHO: World Health Organization
