# Supplementary material for: Reporting Digital Health Implementations Based on the iCHECK-DH Guidelines and Checklist: Development of an Interactive Toolkit
Source: J Med Internet Res. 2025 Aug 8;27:e74235. doi: 10.2196/74235 (PMC12334105; doi:10.2196/74235)
Supplement: Multimedia Appendix 1 [file jmir-v27-e74235-s001.docx]

Supplementary file 1

**Use case report generated by iCHECK- DH toolkit**

**Title: Web based system for stock management of family planning commodities in Sri Lanka: an implementation report**

**Abstract:**

### **Background:** Web based family Planning Stock management system was introduced as a centralised, real-time digital platform to streamline the management of family planning commodity stocks, ensure equitable distribution, and enhance decision-making, replacing the initial paper-based system. It aimed to maintain optimal stock levels of family planning commodities, minimize wastage, and improve redistribution efficiency, while ensuring the timeliness, accuracy, and completeness of stock data reporting.

### **Methods:** The system was developed on a DHIS2-based digital platform integrated with existing systems. Key elements of the intervention included automated data validation, GIS-enabled dashboards for stock level visualisation, and standardised reporting templates. Implementation was phased, starting with Regional Medical Supply Divisions before scaling to the Medical Officer of Health level, Capacity-building measures, including training sessions and user guides, were provided to ensure efficient use of the system.

### **Results:** The percentage of facilities submitting timely and complete reports increased from 84% in 2022 to 86% in 2023 and 90% in 2024, supported by automated data validation features. Real-time dashboards enabled equitable redistribution of commodities, significantly reducing stockouts and overstocking. GIS mapping highlighted regional disparities, further optimizing resource allocation.

**Conclusion:** Implementation successfully addressed key gaps in supply chain management, ensuring uninterrupted service delivery and efficient resource utilisation. This implementation serves as a model for leveraging digital health solutions to enhance health system performance.

**Key Words:** Family planning commodities, web-based, stock management, implementation, resource allocation.

**Introduction**

The Web-Based Family Planning Stock Management System has been implemented to streamline family planning stock management at the national level, involving Family Health Bureau (FHB), Regional Medical Supply Division (RMSD), and Medical Officer of Health (MOH) offices. This report focuses on the system’s initial three years of national implementation, which began in July 2021.

Sri Lanka’s robust public health system, characterized by widespread access to primary health care, provides a strong foundation for this initiative [1]. The Sri Lankan government health system is divided into two main sectors: curative and preventive health [2]. The preventive health sector plays a crucial role in ensuring population-level health outcomes through targeted interventions, such as the Reproductive, Maternal, Newborn, Child, Adolescent, and Youth Health (RMNCAYH) programme [3]. The Family Health Bureau (FHB), functioning under the Ministry of Health, serves as the focal point for the RMNCAYH programme, providing leadership in planning, implementation, monitoring, and evaluation. Family planning, a key pillar of RMNCAYH, is primarily implemented at the Medical Officer of Health (MOH) level, with each MOH area divided into several Public Health Midwife (PHM) areas. PHMs are responsible for delivering a continuum of maternal, newborn, and child health services to the community [4].

**Context:** This implementation specifically addresses the stock management of family planning commodities, a critical component of the family planning programme (see Figure1).


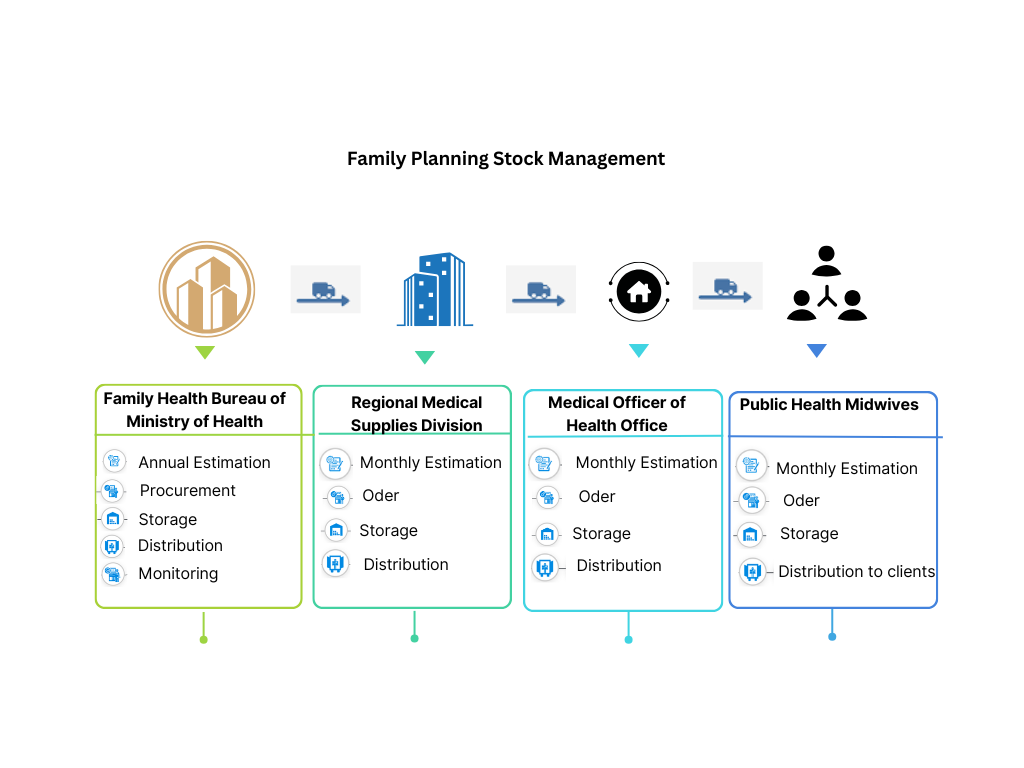


Figure 1: Family planning commodities stock management

Previously, family planning stock management was conducted using a paper-based system. Each month, MOH units were required to submit the H 1158 Family Planning Stock Return to their respective Regional Medical Supplies Divisions (RMSDs). The RMSDs used these returns to formulate district-level monthly stock estimates and subsequently submitted their own H 1158 stock management returns to the Family Health Bureau (FHB). Based on these returns, the FHB issued family planning commodities to RMSDs on a monthly basis, which were then distributed to MOH units and healthcare facilities under their jurisdiction. While this system provided a basic framework for stock management, it had several limitations.

**Problem statement:** The previously used paper-based system was inefficient and made it difficult to track real-time data. This system was highly manual and labour-intensive, providing limited visibility into stock levels in real time. These constraints often resulted in delays in decision-making and inefficiencies in stock redistribution, leading to critical issues such as out-of-stock situations in some areas and overstocking in others, which caused wastage and service disruptions. The absence of a real-time stock management system for family planning commodities posed significant challenges in maintaining optimal stock levels across various levels of care. The health system challenges addressed by the Web-Based Family Planning Stock Management System (WFPSMS) have been classified according to the WHO Classification of Digital Health Intervention (see figure 2)

**2.1 Insufficient supply of**

**commodities**

**6.1 Inadequate workflow management**

**3.2.1 Manage inventory and distribution of health commodities**

**3.2.2 Notify stock levels of health commodities**

**B.6 Logistics management information systems (LMIS)**

**D.5 Geographic information systems (GIS)**

**1.6 Insufficient utilization of data and information**

**Health System Challenges**

**Digital Health Interventions**

**Services and application types**

*Figure 2. Classification of health system challenges, digital health interventions and application types in relation to web-based family planning stock management system [5]*

## The initial paper base system was decided to transform into a web-based system, to centralized, real-time digital platform to streamline inventory management, ensure equitable distribution, and enhance decision-making. It also included GIS mapping to visualize contraceptive availability at all levels on the dashboard enabling detailed monitoring of stock status.

**Similar intervention:**  The web-based family planning stock management system in Bangladesh served as a model for this initiative, showcasing the potential of a web-based platform to improve stock management. Lessons learned from this intervention provided valuable insights for the Sri Lankan implementation, ensuring a contextually relevant adaptation of the system.

**Methods:**

## **Aims and Objectives:** This initiative aimed to address existing gaps in stock management that were evident in the initial paper-based system, ensuring optimal stock levels across all levels of care to support uninterrupted service delivery. The primary outcome was to ensure that healthcare facilities maintained optimal stock levels, avoiding both shortages and wastage due to overstocking. Additionally, it sought to enable the efficient redistribution of stock between facilities to maintain balance. The secondary outcomes included ensuring the timeliness of data submission for proactive stock management, improving the accuracy of data through automation to reduce manual errors in reporting, and achieving data completeness by ensuring that all institutions, including MOH and RMSD levels, consistently report their data for comprehensive system coverage.

### Key performance indicators (KPIs) for this initiative included

### KPI 1. Percentage of facilities maintaining stock levels within predefined minimum and maximum thresholds each month.

KPI 2. Percentage of stockouts reported at any level, monitored monthly.

KPI 3. Percentage of overstock incidents identified and addressed through redistribution.

KPI 4: Number of redistribution requests processed successfully

KPI: Monthly reporting rate

KPI 5: Timely reporting rate (Percentage of facilities submitting stock data by the 5th of each month)

### The system enforces standard reporting through automated calculations, such as predefined minimum and maximum stock levels for each area, ensuring consistent and accurate data entry. Completeness is ensured through a built-in system feature that mandates the submission of fully completed reports. Reports cannot be submitted unless all fields are filled and validated, ensuring that incomplete forms are not accepted. As a result, completeness is always maintained at 100%.

##

## **Blueprint summary**

**Design and development**

The **WFPSMS** conceptualization was guided by a detailed requirement analysis, which included consultations with key stakeholders from the Monitoring and Evaluation(M&E) unit of FHB, family planning unit of FHB, and RMSs, MOHs. Their insights shaped the system’s design to align with operational needs and user expectations, ensuring its relevance and practicality.

The system was designed and developed by a team of information technology professionals and health informatics specialists and consultant community physicians at the FHB. The use of in-house capacities at FHB for the system’s development ensured that it met both technical and operational needs, Digital forms were designed to replicate the structure and sequence of the paper-based stock management format (H1158), ensuring minimal disruption to established workflows and providing familiarity for end-users transitioning to the digital platform. The web-based platform facilitated the submission of monthly stock returns at both district and divisional levels. One of its standout features was the integration of Geographic Information System (GIS) mapping, which enabled real-time visualization of stock levels across national, district, and divisional levels. This feature allowed stakeholders to identify and address areas with stock shortages or surpluses efficiently. National-level data inputs, such as annual estimates for each RMSD and MOH, were incorporated into the system to calculate minimum and maximum standard report amounts.

**Implementation**

One-page user guides related to technical knowledge of WFPSMS in electronic reproductive health management information system (eRHMIS) system and detailed instruction sheets regarding filling of stock returns in WFPSMS were prepared to assist users in navigating the platform. Capacity building was done through comprehensive Two-day online training sessions for RMSD and MOH staff. These sessions were tailored to accommodate Tamil- and Sinhala-speaking users, ensuring inclusivity. Refresher trainings were provided as needed to address any skill gaps and reinforce knowledge.

The system was initially deployed at the RMSD level, targeting primary stakeholders for effective adoption after that it scaled to MOH level. MOHs were already familiar with the web-based system, as they had been using eRHMIS for entering maternal and child health data. This phased approach ensured the system’s capabilities and support mechanisms were well-tested before broader scaling. RMSDs and MOHs were not required to provide additional logistics, such as computers or internet connections, as these have already been supplied by the Ministry of Health.

**Technical design**

The system was selected and customized using the DHIS2 platform, which is a well-established, free, and open-source solution. The platform requires minimal coding and is tailored to meet the requirements of the Family Health Bureau (FHB) through its in-house capacity. Detailed customization documentation and support are available through the Family health Bureau. Further information about DHIS2, including code and resources, can be accessed on the [official DHIS2 website](https://www.dhis2.org/). The DHIS2 platform integrates several tools that cater to the needs of data entry, visualization, and reporting:

- Data Entry Module: Enables systematic and efficient input of stock data.
- Data Visualizer: Provides powerful analysis and visualization capabilities through graphs, tables, and indicators.
- Dashboard: Offers real-time insights through an interactive interface for informed decision-making.
- Reports and Maps: Generates standard reports with GIS-enabled mapping to visualize geographic disparities and stock levels.

The system builds upon the eRHMIS (https://fhb.health.gov.lk/electronic-sysems/,) which was established in 2017. eRHMIS was already in use for data collection and program monitoring at the FHB. By building on the eRHMIS infrastructure, the WFPSMS avoided duplication of efforts, ensured seamless integration, and created a foundation for future scalability.

**Target**

The targeted implementation sites for the WFPSMS included 28 Regional Medical Supply Divisions (RMSDs) and 258 Medical Officer of Health (MOH) offices across Sri Lanka (see figure 3). RMSDs serve as regional hubs for the storage and distribution of family planning commodities. They are equipped with warehousing facilities, trained personnel for inventory management, and logistical resources to support stock redistribution. Each RMSD is staffed by supply chain personnel and overseen by healthcare administrators responsible for regional-level stock monitoring and reporting. MOH offices are the primary points for service delivery and are staffed by public health professionals, including Medical Officers of Health, Public Health Midwives (PHMs). MOH offices are responsible for ensuring the availability of family planning commodities at the community level. The system has currently been implemented at the RMSD and MOH levels.

Expansion to PHM-level tracking will be considered as a future phase when additional resources become available. PHMs are field level health workers who play a critical role in last-mile service delivery and stock management. Including them in subsequent phases would further enhance the granularity and comprehensiveness of the system, enabling even more precise tracking and redistribution of stock.

Hospitals also provide family planning services to clients, but the number of clients for these services are relatively low. Due to practical challenges in integrating hospitals into the web-based system, they continue to manually enter data into H1158 forms and submit them to the RMSD to request their stocks.


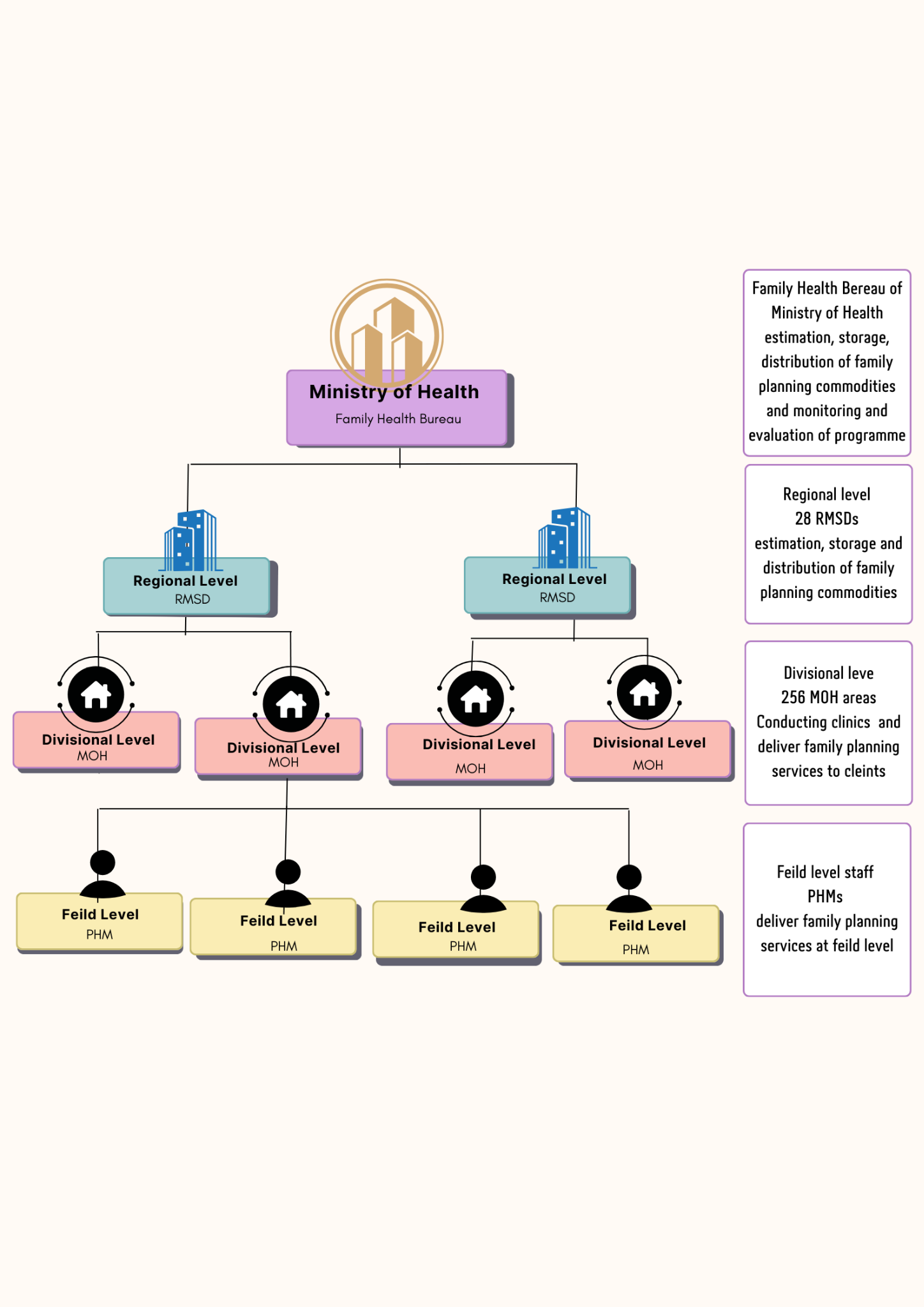


*Figure 3: Targeted implementations sites of web-based family planning stock management system*

## **Data**

WFPSMS follows a structured data life cycle to ensure integrity and compliance. Ownership of the system, including all associated data and intellectual property, resides with the FHB, ensuring centralized management and oversight to align with national health objectives. Instructions for the implementation and use of the system were provided under the guidance of the Director of the Family Health Bureau (FHB).

Data is collected at multiple levels, MOHs are submitting a monthly stock return to relevant RMSDs and RMSDs collect all its MOHs returns and calculate the district requirements and enter monthly stock return into WFPSMS. The system processes data in real time, providing actionable insights, such as demand forecasts and consumption trends, while automating notifications for stock-related actions.


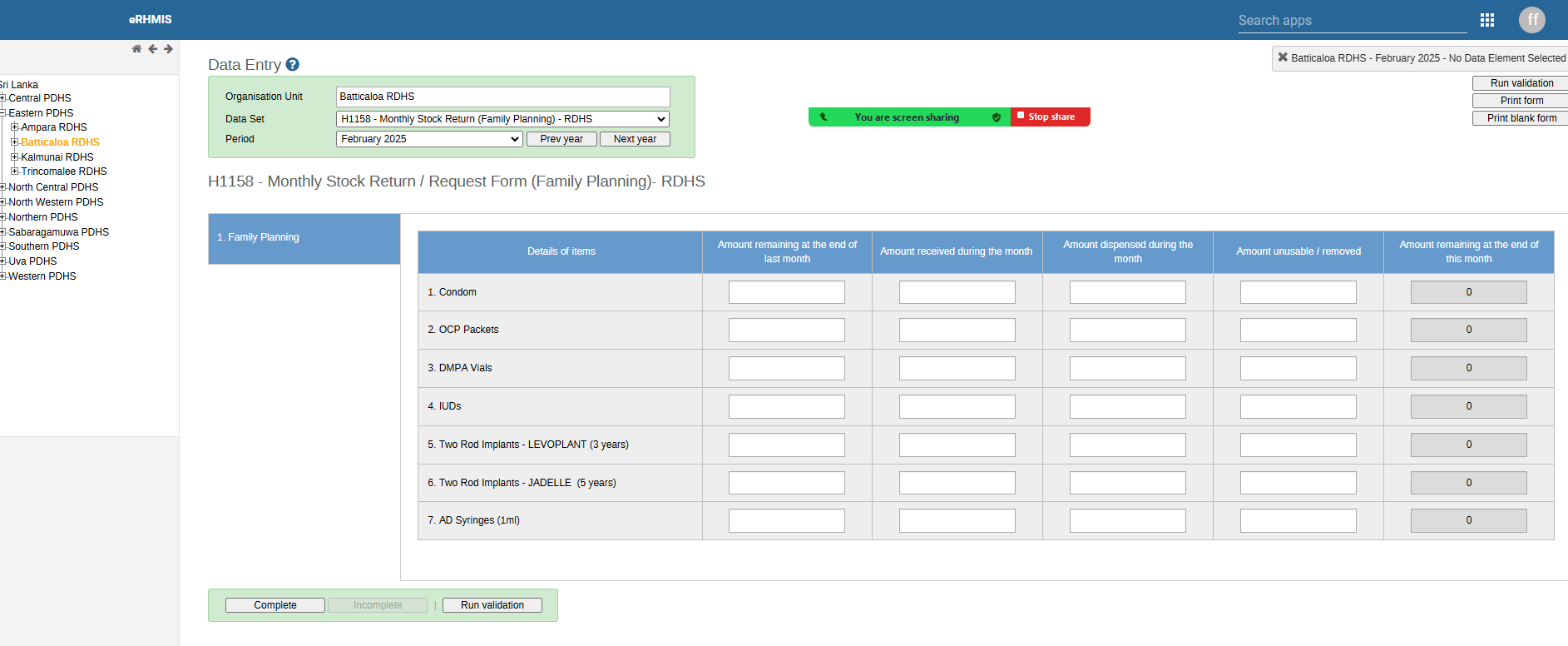


*Figure 4a Web Based data entering form for monthly stock return/request form (district level)*


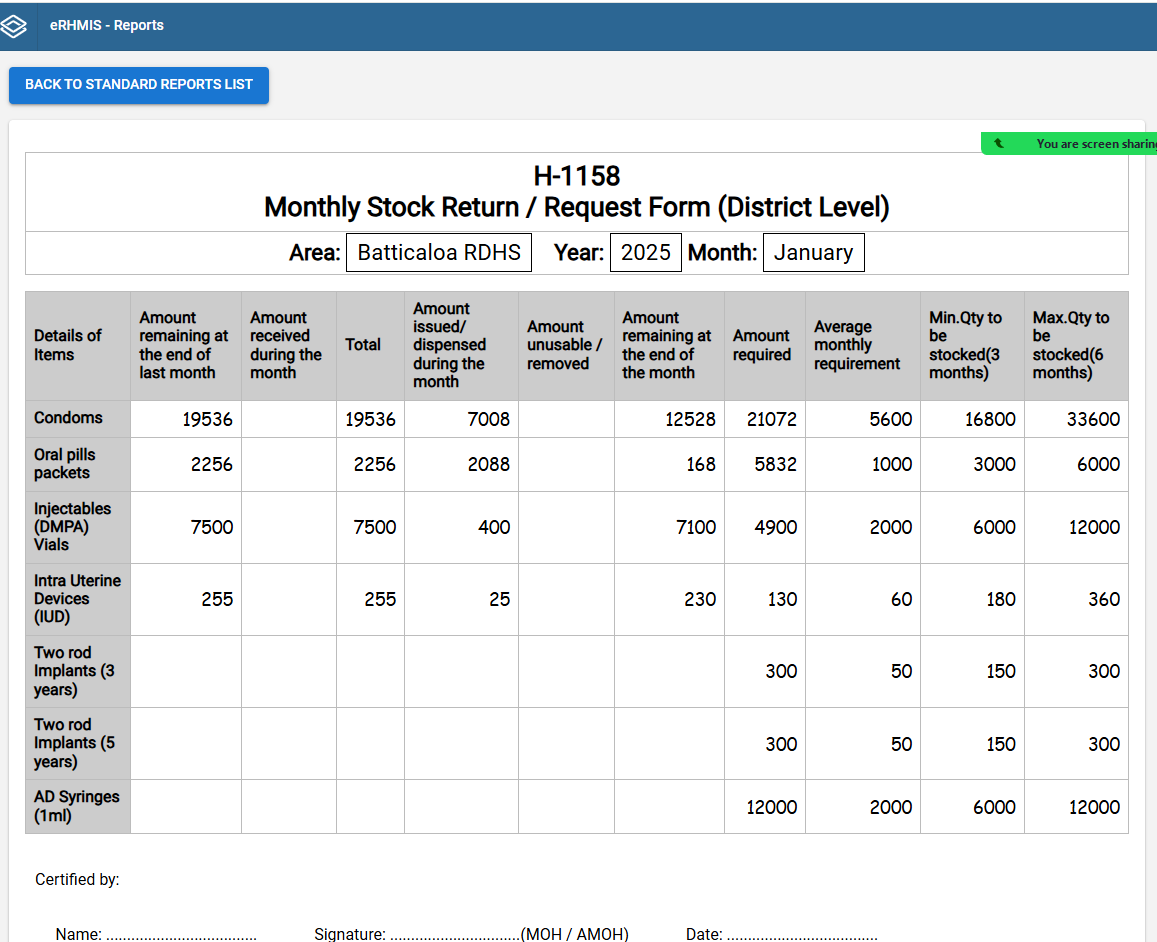


*Figure 4b Monthly stock return (district level) report generated by web-based family planning stock management system*

MOHs and RMSDs are only required to input data on the amounts received during the month and the amounts deemed unusable or removed. All other columns are automated within the system, reducing manual workload and potential errors. The system incorporates a pre-fed formula to calculate the minimum and maximum stock amounts, ensuring standardized and accurate computations for optimal stock management. After entering the data, the person responsible must press the "Complete" button to indicate that data entry is finalized. Once this step is completed, the data undergoes review by the MOH or RMSD in-charge. After their approval, the data is formally submitted to the system. Once submitted, it takes approximately one day for the information to be visualized and accessible at the national level,

The data generated and collected through the system is securely stored on a dedicated server located at the FHB with daily backups. At the national level, data is locked after the first quarter (Q1) of the following year. This ensures data integrity and prevents retrospective modifications, enabling accurate reporting and analysis for the completed year. Authorized personnel, such as the Medical Officer of Health (MOH) or the Officer in Charge at the Regional Medical Supply Division (RMSD), may update data entries upon request from a National Level Consultant to ensure accuracy, if modifications are needed after the data has been locked. Within The system, data is shared with authorized stakeholders at regional and national levels through controlled access, ensuring confidentiality. Outside the system, access to data for sharing purposes must be approved by the program manager, and institutional approval is also required.

Each user of the system is provided with a unique username and password to ensure secure access and accountability. Users are restricted to editing and managing data specific to their own district return, adding an additional layer of data protection. Strict protocols are in place to maintain confidentiality and integrity, including instructions not to share login credentials, even within the same MOH or institution.

The system maintains an audit trail, recording actions such as data entry and deletion, linking them to the responsible user. This ensures transparency and accountability for all operations. In the event of personnel changes, such as the appointment of a new MOH, a new username must be issued. Additionally, users are required to change their password after being issued by the national unit for added security. If an individual leaves their role, their account is deactivated to prevent unauthorized access, maintaining the overall security and reliability of the system.

The system allows data entry at the MOH and RMSD levels, with data visualized at higher levels through thematic layers in map applications, enabling RMSD to view specific MOHs data and the Family Health Bureau (FHB) to access data from all levels.

The system architecture includes multiple modules within the eRHMIS platform, such as HPB (Health Promotion Bureau) and EOFS (Environment Occupation and Food Safety unit), each with separate components. This compartmentalization ensures that users can access only the data relevant to their specific role and module, thereby maintaining data segregation and security.

The data server is protected with stringent measures. Only the Monitoring and Evaluation (M&E) unit of FHB has the authority to make changes in the system, such as modifying data. Other users are limited to analysing and visualizing data, preventing unauthorized alterations.

The system ensures confidentiality by not collecting individual-level data. While users can analyse and visualize the aggregated data, there is no integration with other systems. There are no existing cross-border data-sharing agreements. The implementation aligns with the applicable legal frameworks, including the Right to Information (RTI) Act. Under this act, data must be provided when requested, ensuring transparency while adhering to legal obligations.

#### **Interoperability**

The WFFSMS currently operates as a standalone platform without direct connectivity or integration with other systems. However, future plans aim to enable interoperability to enhance data exchange and system functionality. Proposed features include integrating the WFPSMS with Channel, the inventory management software used at the Family Health Bureau. Currently, family planning commodities, instruments, and equipment are recorded in Channel upon receipt and issued to RMSDs through the same system. Connecting the two platforms will enable seamless data exchange, improving stock tracking, minimising discrepancies, and ensuring a more efficient supply chain for family planning services nationwide. Future plans could include integration with hospital EMR systems, the National Electronic Health Record (NEHR), and Personal Health Number (PHN) systems to streamline healthcare data management further.

###

### **Participating Entities**

The WFPSMS is a government initiative and the key partners in the implementation included the M&E Unit of the Family Planning unit of FHB, which led requirements gathering and system customization, and RMSD and MOH Units, which contributed to workflow definitions and user input. The project was fully financed by the Government of Sri Lanka, covering all phases from development to operations. The system and its intellectual property are owned by the FHB, ensuring long-term sustainability as a public resource with plans for future upgrades to meet evolving family planning stock management needs.

## **Item 13: Budget Planning (M)**

##

## No budget was required for the development of the intervention, as it was created using the in-house capabilities of the Family Health Bureau (FHB). The team utilized existing resources and expertise, with no external costs for development, and a free open-source DHIS2 platform to develop intervention.

The implementation included a two-day training program for RMSD personnel, which was funded by the Government of Sri Lanka (GoSL). There were no additional costs for maintaining the intervention technology or ownership, as these were managed using existing inhouse resources of FHB.

##

##

## **Sustainability**

The WFPSMS leverages the open-source DHIS2 platform, eliminating licensing costs and reducing upfront expenses. Financial sustainability is ensured through government ownership under the Family Health Bureau (FHB), which maintains in-house capacity for system customization and maintenance. Additionally, the system will be supported by allocating a portion of the annual health budget for server upkeep, software updates, and user training. By optimizing stock distribution and minimizing wastage, such as avoiding shortages and overstock, the system contributes to significant cost savings, further enhancing its financial viability.

To ensure effective and sustained use, the FHB has implemented a user support system that includes refresher training programs for RMSD and MOH staff. These programs ensure continuous user engagement and skill enhancement, enabling stakeholders to make the most of the system’s features. Additionally, comprehensive user guides and instructional materials provide self-sufficiency in system use, with updates to reflect any changes or enhancements. Promoting data use at all levels is a central focus of the sustainability model. While initially designed for national-level reporting, the system’s dashboards, GIS maps, and visual analytics empower users at the RMSD and MOH levels to monitor trends and make data-driven decisions. The system was intentionally designed to align closely with existing paper records, preserving the structure and order of columns to ensure ease of adoption. Future updates to the paper-based system can be mirrored in the digital platform, ensuring continued compatibility and smooth transitions.

The system will be updated to meet changing requirements, such as evolving policies or health priorities, while regular server upgrades will ensure scalability and functionality. The institutionalization of the system as part of the national digital health strategy further supports its long-term success. Exit strategies are built into the sustainability model. Embedding the system into routine FHB operations ensures its integration as a critical tool for family planning management.

**Results:**

**Coverage**

WFPSMS has been implemented at the national level in Sri Lanka, covering all District Regional Medical Supplies Divisions (RMSDs) and Medical Officer of Health (MOH) areas. This comprehensive coverage ensures that the system is accessible to all administrative regions responsible for family planning stock management across the country.

##

## **Outcome**

The implementation of the WFPSMS has yielded significant primary and secondary outcomes, aligned with predefined measures.

#### Primary Outcomes: The system has enabled efficient management of family planning commodities, ensuring that minimum and maximum stock levels are maintained across RMSD and MOH levels. Timely identification of low-stock regions allows for the redistribution of resources, minimizing wastage and preventing shortages that could disrupt services.


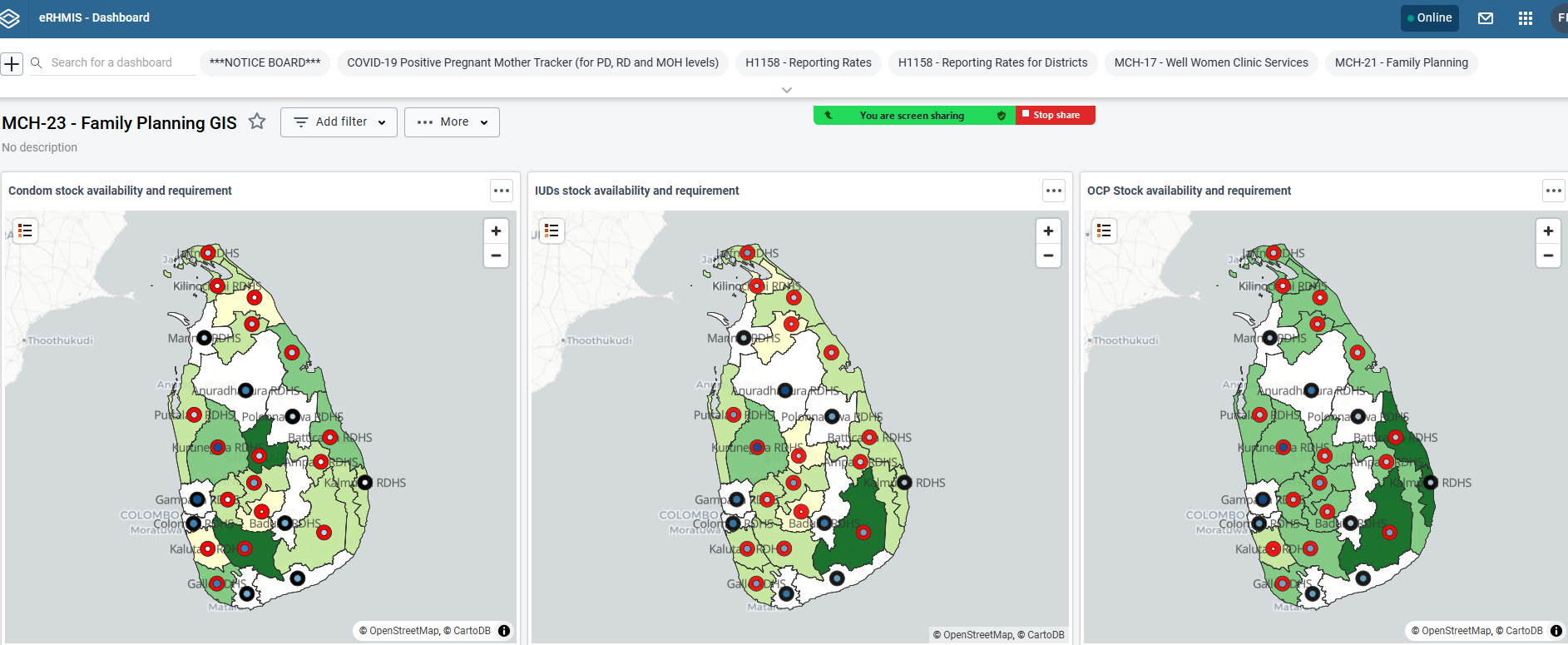


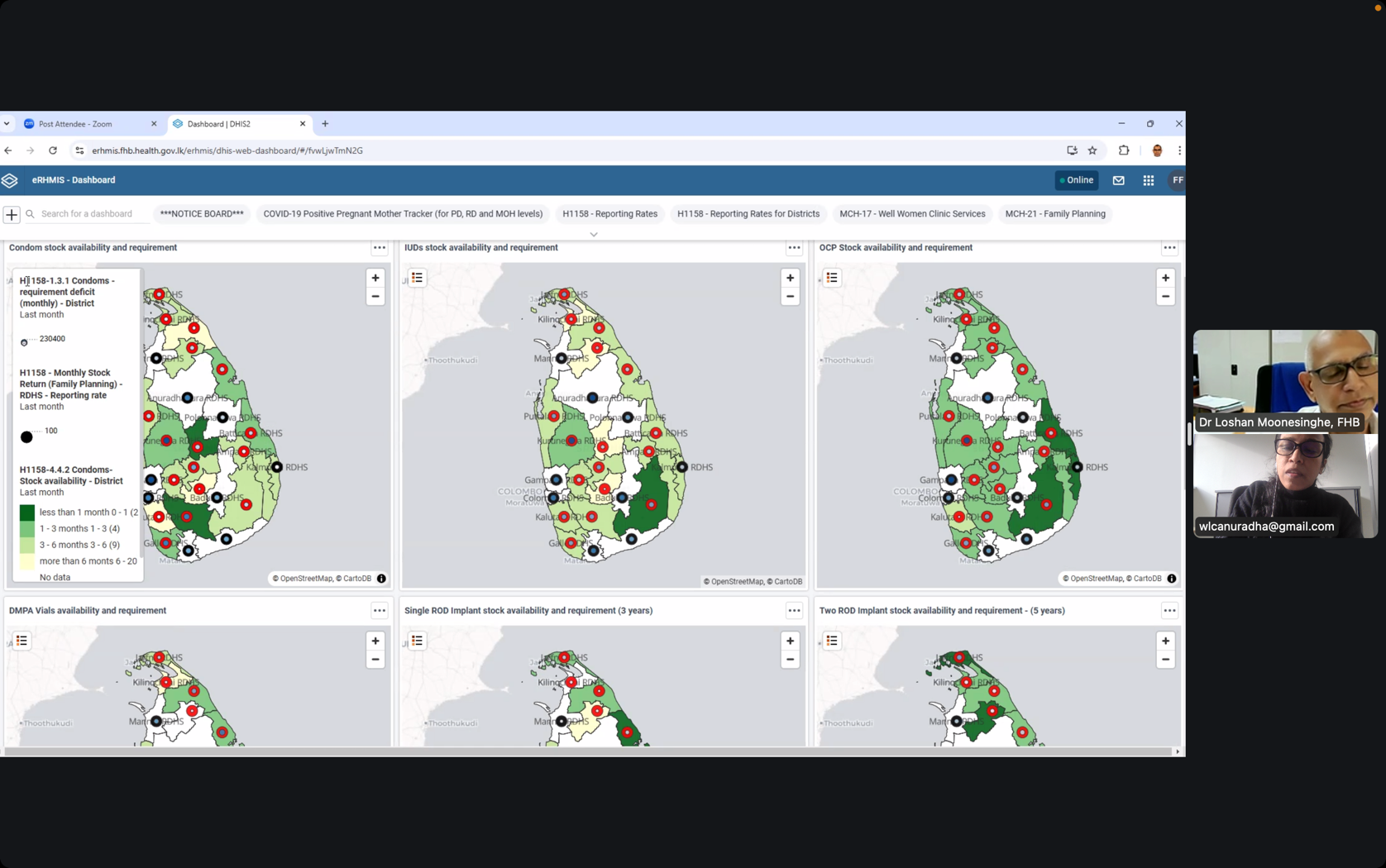


### Figure 5 GIS mapping of family planning commodities availability of districts according to stock levels within predefined minimum and maximum thresholds for the month of January 2025

Monthly reporting rates have improved, with data submission monitored before the 5th of each month. This successful implementation had a reporting rate of monthly stock return as 84.2%, 86.6% and 90. 1% at the national level in 2022, 2023, 2024 with 21 RMSDs achieving 100%. Reporting rate of the Annual Estimation at RMSDs was 100% in 2022 and 2023 and 2024


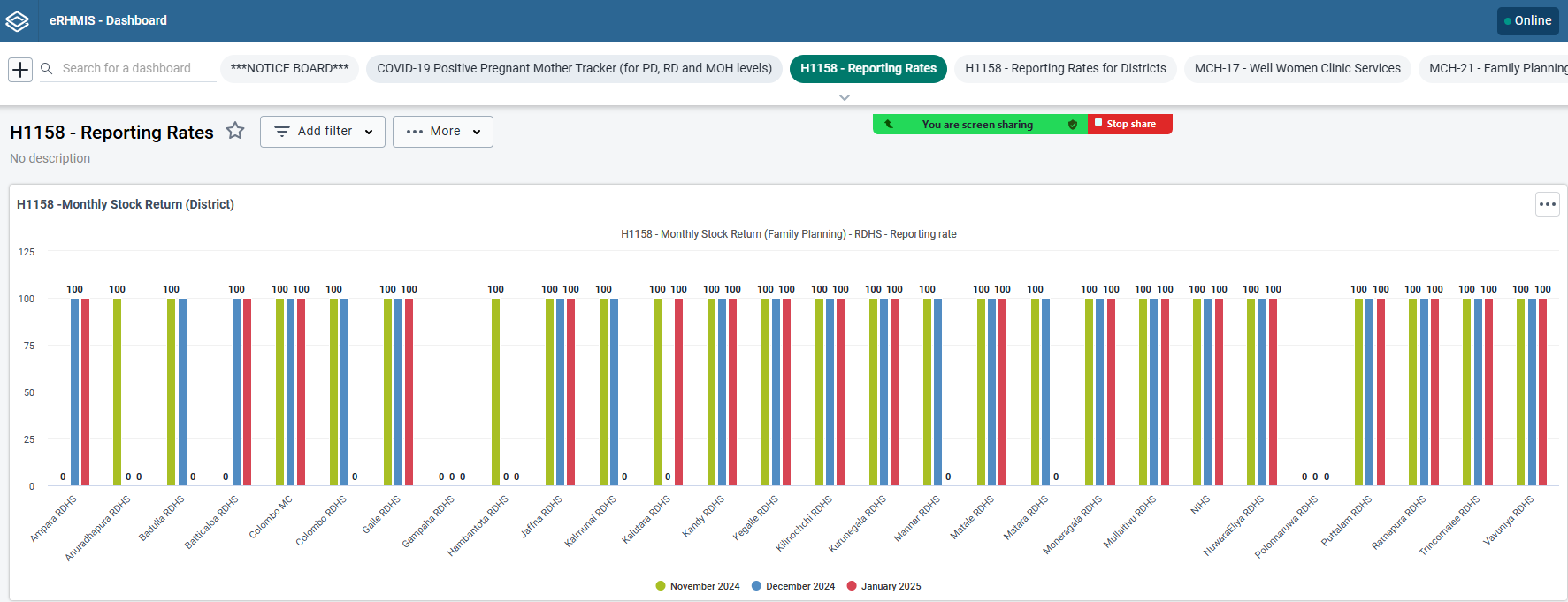


Figure 6a Monthly stock return (family planning) timely reporting rate by district for November, December 2024vand January 2025


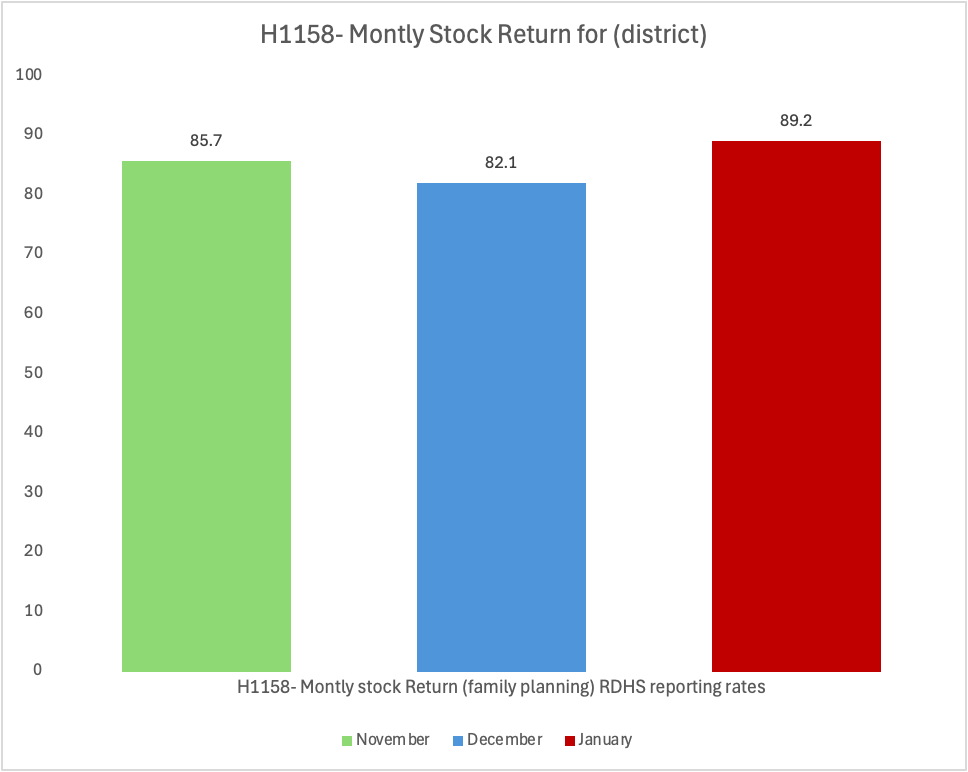


Figure 6b Monthly stock return (family planning) timely reporting rate of all districts in Sri Lanka for November, December 2024 and January 2025

This enhancement ensures that stock levels are consistently updated, and actionable insights are available in real-time.

Automated processes in data entry and reporting have significantly reduced errors. Reporting completeness is tracked at the MOH and RMSD levels, ensuring that all institutions contribute to the dataset for comprehensive analysis.

**Success Factors**
The success of the Web-Based Family Planning Stock Management System can be attributed to several key factors. Stakeholder engagement played a crucial role, with early involvement of the Ministry of Health, regional health administrators, and healthcare facility staff ensuring that the system aligned with user needs. Regular communication with stakeholders fostered a sense of ownership, facilitating smoother adoption. Capacity building through comprehensive training programs significantly contributed to the system’s success, with hands-on, practical training leading to high levels of staff satisfaction. Additionally, the initiative benefitted from a supportive policy environment, aligning with Sri Lanka’s national digital health strategy and recent legislation promoting digital solutions in public health. The system’s scalable design, which integrated seamlessly with the existing DHIS2-based eRHMIS infrastructure, optimized resource utilization and minimized duplication. Finally, the adoption of open-source technology reduced software costs and enabled the platform to be customized to local requirements.

**Challenges to Implementation**
Despite its success, the project encountered several challenges. Resistance to change among staff, particularly in rural facilities, necessitated additional training and consistent technical support to address their unfamiliarity with digital tools. Infrastructure limitations, such as limited internet connectivity and outdated hardware in rural areas, delayed full implementation, highlighting the need for reliable infrastructure. Technical issues, including occasional software glitches and updates, caused minor disruptions during the early phases, which were mitigated by a dedicated technical support team. Additionally, data integration challenges, including the lack of interoperability with the channel inventory management system at FHB, limited the system’s full potential.

One significant challenge is that the system has not yet been scaled up to the Public Health Midwife (PHM) level. Although stock redistributions from the MOH level to PHMs are carried out, the lack of data entry at the PHM level limits the system’s ability to track final distribution and utilization at the community level. Expanding the system to include PHM-level data entry would require considerable effort in training PHM staff and a significant investment of resources. Given the current financial and operational constraints, the implementation is limited to the RMSD and MOH levels. However, scaling up to the PHM level can be considered in a future phase when additional resources are available.

**Unintended Consequences**The implementation was influenced by external factors. On the positive side, the COVID-19 pandemic underscored the need for efficient digital health solutions, accelerating buy-in from stakeholders and policymakers. Conversely, fluctuating economic conditions during implementation delayed the procurement of essential resources, such as hardware and software licenses.

**Recommendations for Future Implementations**Future implementations should begin with a thorough needs assessment to identify and address infrastructure gaps before rollout. Regular stakeholder consultations should be prioritized to address data governance concerns and foster collaboration. A contingency budget should be allocated to manage unexpected delays or technical issues. For regions with limited internet connectivity, developing offline capabilities is essential. Finally, continuous monitoring and evaluation of the system will help address emerging challenges and ensure scalability. By learning from these experiences, future initiatives can build on the success of this system to achieve an even greater impact.

**Discussion**

The implementation of the WFPSMS marks a significant advancement in enhancing healthcare delivery and resource management in Sri Lanka. This system has effectively addressed critical challenges, such as stock shortages and overstocking, by ensuring optimal stock levels and enabling timely redistribution. It has also contributed to operational efficiency, reducing waste and ensuring equitable resource distribution across healthcare facilities. Furthermore, the integration of GIS mapping and data visualization tools has empowered stakeholders with real-time insights, facilitating evidence-based decision-making at both regional and national levels.

The project’s success is grounded in several key achievements. The system optimized stock distribution processes, prevented inefficiencies, and ensured timeliness in reporting, improving accuracy and completeness at both RMSD and MOH levels. Leveraging the open-source DHIS2 platform has proven cost-effective, scalable, and customizable to the evolving needs of the healthcare sector. The implementation has also enhanced reporting quality, enabling more reliable resource planning and management.

Looking ahead, this intervention sets a strong foundation for further scalability and interoperability within Sri Lanka’s digital health system. Future plans include integration with hospital EMR systems and the Channel system, in which family planning commodities, instruments, and equipment are recorded upon receipt and issued to RMSDs.

Additionally, the project highlights the importance of policy and advocacy to sustain digital health initiatives. Continued government commitment, supportive legislation, and investment in infrastructure will be essential to ensuring long-term success. Promoting a culture of data-driven decision-making within healthcare institutions will further enhance the system’s impact.

The success of this project offers a replicable model for other countries facing similar challenges in stock management, particularly in resource-limited settings. By refining and expanding this system, Sri Lanka can strengthen its healthcare infrastructure, improve service delivery, and position itself as a regional leader in digital health transformation.

In conclusion, the project underscores the value of leveraging digital health tools to address systemic inefficiencies in healthcare. By continuing to refine and expand the system, Sri Lanka can further strengthen its healthcare infrastructure, improve service delivery, and serve as a leader in the digital health transformation within the region.

**General:**

No conflicts of interest were identified in the course of the implementation. The intervention was funded and fully supported by the Government of Sri Lanka (GOSL), ensuring transparency and alignment with public health goals.

**References**

1. Rajapaksa L, Silva PD, Abeykoon P, Somatunga L, Sathasivam S, Perera S, Fernando E, Silva DD, Perera A, Perera U, Weerasekara Y. Sri Lanka health system review. InSri Lanka health system review 2021.
2. Jayasekara RS, Schultz T. Health status, trends, and issues in Sri Lanka. Nursing & health sciences. 2007 Sep;9(3):228-33.
3. De Silva C, Jayakody H. Essential service delivery in reproductive, maternal and child health services during the pandemic. Journal of the College of Community Physicians of Sri Lanka. 2020 Dec 31;26(4).
4. Adikari PS, Pathirathna KG, Kumarawansa WK, Koggalage PD. Role of MOH as a grassroots public health manager in preparedness and response for COVID-19 pandemic in Sri Lanka. AIMS public health. 2020 Aug 5;7(3):606.
5. World Health Organization (2023) Classification of digital interventions, services and applications in health. <https://www.who.int/publications/i/item/9789240081949>. Accessed 17 June 2024
